# Purging to Prevent Traumatic Inflammation

**Published:** 1853-05

**Authors:** 


*Purging, to prevent Traumatic Inflammation.*—Prof. Mettauer, in the *Virginia Medical and Surgical Journal*, has an interesting paper on the prophylaxis of traumatic inflammation, from which we copy the following:

"In support of the efficacy of early purging, after capital surgical operations, in preventing traumatic inflammation, I will state that I have operated for stone in the bladder seventy-nine times, and out of this number I purged seventy-five of the patients in from five to twelve hours after the operation, and in not a solitary instance did inflammation occur in any degree beyond what was neccessary for the healing of the wound. In some of these cases the calculi were of such large size as to require to be crushed before they could be extracted, while others were large enough to render extraction difficult, in either case neccessarily subjecting the bladder to more or less injury. The only cases I have lost were the four I did not purge. I have from this statement, prevented traumatic inflammation in seventy-five lithotomy patients by early purging, all of whom experienced rapid recoveries; while I have lost four who were not purged.

"I have operated for ascites fifty-eight times, and some of the serous accumulations were very large, one exceeding ten gallons. I purged in every case from five to twelve hours after the operation, without inflammation succeeding in but a solitary instance; and in this, paracentesis seemed to aggravate the pre-existing phlogosis induced by catarrh. I have purged in a few hours after reducing strangulated hernia in thirty cases, and succeeded in preventing inflammation in every instance. In fourteen cases of vesico-vaginal fistula, for which I have successfully operated, I was enabled by early purging to avert inflammation in every instance and some of the cases were very bad ones, requiring that the bladder should be extensively irritated by the operation. I have operated for more than two hundred cases of stricture, and by early purging undue inflammation was prevented in every instance, and perfect cures followed. Besides the cases here referred to, I have, in hundreds of instances, purged early after severe surgical operations, and uniformly with the effect of averting undue degrees of inflammation, or of preventing phlogosis altogether. It is true other measures were also employed in many instances, in connection or alternation with purging, but they were of secondary importance, and merely used as auxiliaries or as temporizers."

